# Systematic Analysis of DNA Demethylase Gene Families in Foxtail Millet (*Setaria italica* L.) and Their Expression Variations after Abiotic Stresses

**DOI:** 10.3390/ijms25084464

**Published:** 2024-04-18

**Authors:** Yingying Sun, Xin Wang, Yunfei Di, Jinxiu Li, Keyu Li, Huanhuan Wei, Fan Zhang, Zhenxia Su

**Affiliations:** 1 College of Life Sciences, Shanxi University, Taiyuan 030006, China; sunyingying95@163.com (Y.S.); 15536361452@163.com (X.W.); 15513999700@163.com (Y.D.); 19581553488@163.com (J.L.); likeyu_stayreal@163.com (K.L.); 17326415531@163.com (H.W.); zf17836588971@163.com (F.Z.); 2 Xinghuacun College (Shanxi Institute of Brewing Technology and Industry), Shanxi University, Taiyuan 030006, China

**Keywords:** *Setaria italica* L., DNA demethylase, abiotic stress, systematic analysis

## Abstract

DNA methylation is a highly conserved epigenetic modification involved in many biological processes, including growth and development, stress response, and secondary metabolism. DNA demethylase (DNA-deMTase) genes have been identified in some plant species; however, there are no reports on the identification and analysis of DNA-deMTase genes in Foxtail millet (*Setaria italica* L.). In this study, seven DNA-deMTases were identified in *S. italica*. These DNA-deMTase genes were divided into four subfamilies (*DML5*, *DML4*, *DML3*, and *ROS1*) by phylogenetic and gene structure analysis. Further analysis shows that the physical and chemical properties of these DNA-deMTases proteins are similar, contain the typical conserved domains of ENCO3c and are located in the nucleus. Furthermore, multiple cis-acting elements were observed in DNA-deMTases, including light responsiveness, phytohormone responsiveness, stress responsiveness, and elements related to plant growth and development. The DNA-deMTase genes are expressed in all tissues detected with certain tissue specificity. Then, we investigated the abundance of DNA-deMTase transcripts under abiotic stresses (cold, drought, salt, ABA, and MeJA). The results showed that different genes of DNA-deMTases were involved in the regulation of different abiotic stresses. In total, our findings will provide a basis for the roles of DNA-deMTase in response to abiotic stress.

## 1. Introduction

DNA methylation is a prominent epigenetic modification in many eukaryotes [1,2,3,4]. Research has shown that epigenetics is the study of heritable phenotypic changes that do not involve changes in the DNA sequence [5], and epigenetic mechanisms regulate critical agronomic traits in crops via DNA methylation, histone modifications, and small RNAs that affect gene expression and impact growth, seeding, germination, and fruit development [6]. DNA methylation is involved in transposons [7,8,9,10], the suppression of gene silencing, genomic imprinting [11], cell differentiation [12], etc., and is a necessary set up for the normal growth and development of organisms. DNA methylation has been shown to be a dynamic process that can be regulated according to different development periods or environmental conditions [13,14], thereby reducing the stress of plant survival in harsh environments and improving the ability of plants to cope with stress [15,16]. The reversibility of DNA methylation allows for the rapid and reversible modification of plant genomic DNA, avoiding excessive gene recombination and population diversity [17], thereby regulating gene expression and maintaining growth and development [18,19,20,21].

The precise status of DNA methylation depends on the function of DNA methyltransferase and DNA demethylase [22]. DNA methylation is catalysed by DNA methyltransferases and occurs by the addition of a methyl group to the C-5 site of cytosine, the N-6 site of adenine, and the N-7 site of guanine [23]. DNA demethylases contain a conserved DNA glycosilase motif and play a critical role in DNA demethylation by excision of the 5-methylcytosine in CG, CHG and CHH (where H stands for A, C or T) sequence contexts [24]. Increasing evidence shows that plant DNA methylation and demethylation are closely associated with various environmental stresses, including cold, drought, heat, heavy metal, salt, and ultraviolet stresses [25,26,27,28,29,30]. Under conditions of stress, the plant genome can overcome the limitation of genome instability through DNA methylation by rapid modification to maintain plant growth and development and evolutionary processes [31,32]. It was shown that abiotic stresses such as water deficit, cold, and salt stress induced demethylation of the repeats in the promoters of ACD6, ACO3, and GSTF14 and transcriptionally activated their expression [33]. It was also shown that the proportion of DNA methylated cytosines in Populus trichocarpa under drought stress was 10.04%, whereas it was only 7.75% in well-watered treatments [34]. Therefore, DNA methylation is considered the molecular response mechanism of plants in the face of adverse stress [8,9,31]. DNA demethylase is a bifunctional enzyme with glycosylase and depurine cleavage enzyme activities during active demethylation [35]. Currently, six DNA demethylases have been identified in plants, including transcriptional activator demeter (*DME*), Repressor of silencing 1 (*ROS1*), Demeter-like protein 2 (*DML2*), Demeter-like protein 3 (*DML3*), Demeter-like protein 4 (*DML4*) and Demeter-like protein 5 (*DML5*) [36].

Foxtail millet (*Setaria italica* [L.] P. Beauv), widely grown in China, Russia, India, and Africa, is one of the most important of ancient cultivated cereal crops. It was domesticated in northern China approximately 11,500 years ago [37,38]. As a C4 crop, *S. italica* is often used as an important model species for stress biology research [39,40,41]. DNA demethylase has already been identified and characterized in several plant species, including *Arabidopsis thaliana*, *Oryza sativa*, *Daucus carota*, *Hordeum vulgare*, *Zea mays*, *Lycopersicon esculentum*, tobacco, legumes and other plants [42,43,44,45,46,47,48,49]. However, no research has yet focused on the identification and analysis of DNA demethylase genes in *S. italica*, based on genome-wide analyses. Therefore, in this study, we performed a comprehensive analysis of *S. italica* DNA demethylase by genome-wide screening and identification, including phylogenetic analysis, gene structure analysis, conserved motifs construction, chromosomal location, and promoter sequence analysis. The expression levels of *S. italica* DNA demethylases under abiotic stresses such as salt, cold, and drought were also studied by qRT-PCR. These data will provide a valuable reference for further studies of DNA demethylase cascades.

## 2. Results

### 2.1. Genome-Wide Identification and Structural Analysis of DNA-deMTase Genes

Blast analysis of reported *A. thaliana*, *O. sativa* and *T. aestivum* DNA-deMTase proteins against the whole genome of *S. italica*. Subsequently, the sequences were aligned, redundant sequences were removed and combined, and Pfam and SMART structural domain searches were used to check whether the candidate genes contained ENCO3c domains, and a total of 7 candidate genes were identified in the *S. italica* genome (Table 1). The DNA-deMTase genes (*SiDML5*, *SiDML3a*, *SiROS1b*, *SiROS1a*, *SiROS1d*, *SiDML3b*, and *SiDML4*) code proteins composed of 286 to 2082 amino acids. The MW ranges from 31.52 to 230.61 kDa. The pI varies from 5.97 to 9.67, and the mean value was 7.43 which predicted that most of them were neutral proteins. The instability index of the DNA-deMTases proteins was greater than 40, and they were unstable proteins; GRAVY analysis showed that all the DNA-deMTase proteins were hydrophilic proteins. The prediction of subcellular localization showed that all DNA-deMTase proteins were localized in the nucleus, and *SiDML5* was also localized in the chloroplast nucleus. Furthermore, the coding regions of DNA-deMTase genes were interrupted by 4 to 19 introns.

### 2.2. Phylogenetic Tree Construction of DNA-deMTases Gene Family Proteins and Analysis of Conserved Structural Domains

**Figure 1:**
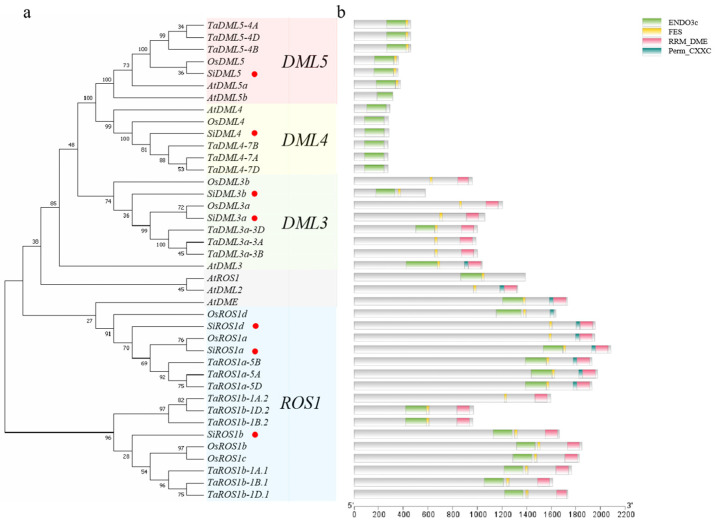
Phylogenetic trees (**a**) and conserved domain (**b**) analysis of DNA demethylase (DNA-deMTase). The red dots in the figure are the DNA-deMTase genes from *S. italica*.

To reveal the evolutionary relationship of the DNA-dMTase genes in *S. italica*, a phylogenetic tree was constructed by comparing the protein sequences of the seven *S. italica* DNA-deMTase genes with those of the seven known *A. thaliana* DNA-deMTase genes, eight *O. sativa* DNA-deMTase genes, and eighteen *T. aestivum* DNA-deMTase genes, for a total of 40 DNA-deMTase genes using the maximum likelihood method. Based on the classification of *O. sativa* DNA-deMTases, the seven DNA-deMTases in cereals were classified into four subfamilies, including *ROS1*, *DML3*, *DML4*, and *DML5*. Among them, *ROS1* was the most abundant, containing three DNA-deMTase genes, *DML3* contained two DNA-deMTase genes, and there was one in each of the *DML4* and *DML5* subfamilies (Figure 1a). CDD and SMART were used to analyze the conserved domains of DNA-deMTases, and the results showed that all DNA-deMTases contained ENCO3c domain. The ENCO3c domains are DNA-deMTase-specific domains that perform the function of glycosidases. *DML5* contains two domains, ENCO3c and FES; *DML4* contains only one ENCO3c domain and *ROS1* and *DML3* subfamilies contain four common conserved domains: ENCO3c, FES, RRM_DME, and Perm-CXXC (Figure 1b).

### 2.3. Observation of Conserved Motif and Gene Structure Analysis of DNA-deMTases in S. italica

**Figure 2:**
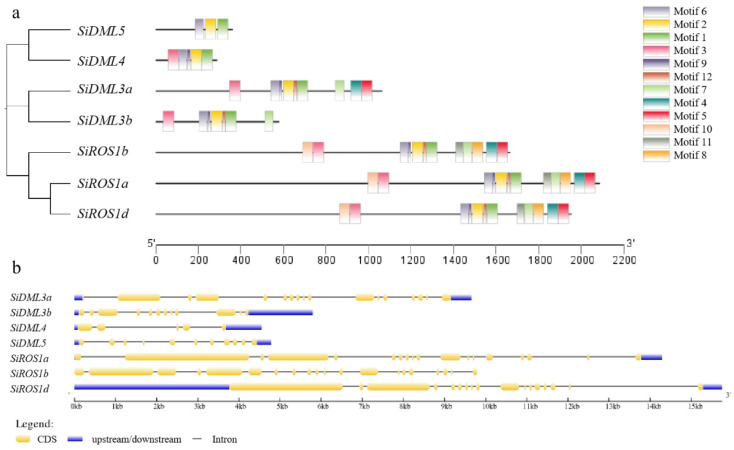
Conserved motifs (**a**) and gene structure (**b**) of DNA-deMTase genes in Foxtail millet *(Setaria italica* L.).

Genetic structure analysis is an important strategy to study genetic evolution. In this study, preliminary Pfam analysis results of the entire predicted proteins were applied to the MEME motif search tool. This analysis was used to further identify conserved motifs in the corresponding conserved domains of all seven DNA-deMTase proteins encoded by this gene family from *S. italica*. The search was performed for all predicted proteins and 12 conserved motifs were identified, but different subfamilies possess different motifs, consistent with their conserved domains. For example, the motifs of the *ROS1* subfamily have the same composition and contain 12 conserved motifs; the *DML3* subfamily contains nine motifs, including motifs 1, 2, 3, 4, 5, 6, 7, 9 and 10; the *DML4* subfamily contains nine motifs, including motifs 1, 2, 3, 6 and 9; the *DML5* subfamily contains nine motifs, including motifs 1, 2 and 6. (Figure 2a). Subsequently, an exon-intron structure analysis was performed to support the phylogeny reconstruction. The schematic structures revealed that each coding sequence of DNA-deMTase gene is disrupted by one or more exons. The DNA-deMTase genes within the same groups of the phylogenetic tree all showed similar exon-intron structures (Figure 2b).

### 2.4. Analysis of Cis-Acting Elements in DNA-deMTase Genes

**Figure 3:**
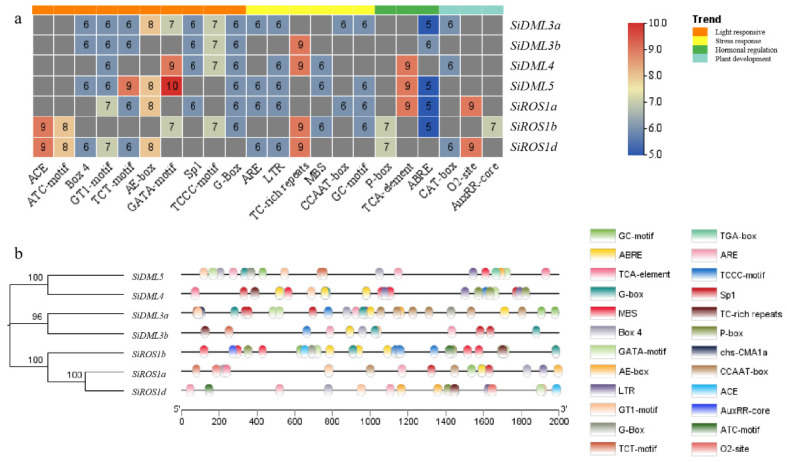
Cis-regulatory elements (CREs) of DNA-deMTase genes in *S. italica.* (**a**): Heatmap showing the number of CREs belonging to the following four categories per DNA-deMTase gene. (**b**): The distribution of different CREs in the promoter region (2000 bp) of DNA-deMTase genes in *S. italica*.

Using the plant CARE database to analyze the 2000 bp cis-acting regulatory elements (CREs) upstream of seven DNA-deMTase genes, we found that the promoter of dMTase in *S. italica* contains types of CREs, including light response (17.5%), stress response (9.8%), hormone regulation (6%) and plant development (1.3%) (Figure 3). The DNA-deMTase promoter has various stress-responsive regulatory elements, such as the LTR involved in low-temperature stress, the MBS involved in drought induction, the TC-rich repeats involved in defense and stress responses, and the ARE regulatory element in response to anaerobic induction. There are also regulatory elements related to hormone signaling, such as the ABRE involved in the abscisic acid response, the P-box involved in the gibberellin response, and the TCA-element involved in the salicylic acid response. In addition, a number of tissue-specific cis-regulatory elements have been identified, such as the GCN4-motif involved in the GCN4-motif required for endosperm-specific expression, and AACA-motif involved in negative endosperm regulation, and AACA-motif associated with negative endosperm regulation. The motif required for endosperm-specific expression and AACA-motif associated with negative endosperm regulation, as well as the CAT-box associated with phloem-specific activation. In conclusion, the presence of multiple CREs in *S. italica* suggests that DNA-deMTase genes can be involved in growth and development, as in the stress response in *S. italica*.

### 2.5. Chromosomal Location and Synteny Analysis

**Figure 4:**
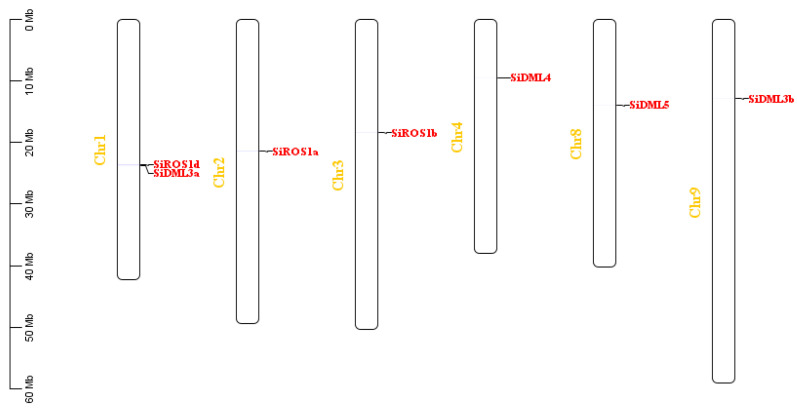
Chromosomal locations of *S. italica* DNA-deMTase genes on 6 chromosomes. The gene names on the right side of each chromosome correspond to the approximate locations of each DNA-deMTase gene.

The distribution of genes on chromosomes provides an important basis for studying the evolution and function of gene families. Combining the chromosomal information of the four *S. italica* genomes and the position of each DNA-deMTase gene on the chromosome, the distribution map of the *S. italica* demethylase gene on the chromosome was obtained (Figure 4). The seven identified genes were dispersed unevenly through nine chromosomes. Chromosome-1 contains two genes (*SiROS1d* and *SiDML3a*), whereas chromosomes 5, 7 and 8 did not contain any target genes, and the other chromosomes have one gene. Most of the DNA-deMTase genes are located in the middle of the chromosomes.

### 2.6. Expression Patterns of DNA-deMTase Genes in S. italica

**Figure 5:**
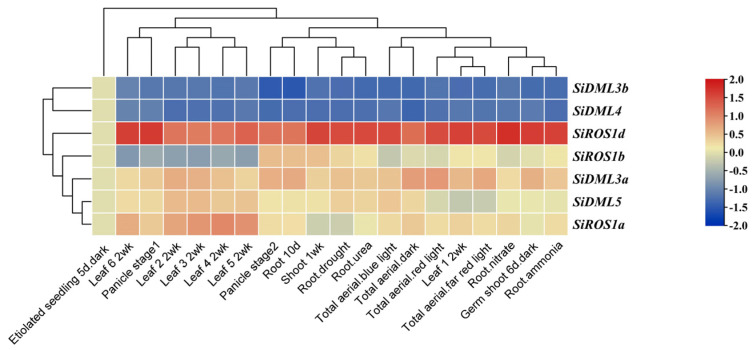
Heat map of *S. italica* DNA-deMTase families’ genes expression patterns across tissues. Color scale bar at the right of heat map represents log2-transformed FPKM (Fragments Per Kilobase of exon per Million fragments mapped) values for each gene, with warmer colors denoting higher expression.

To explore the expression pattern of the DNA-deMTase gene in different tissues in *S. italica*, publicly available Foxtail millet RNA-seq data (GeneAtlas v1 Tissue Sample from Phytozome 13) was used. This dataset included information from various tissues, including etiolated seedling, leaf, panicle, root, shoot, total aerial and germ shoot (Figure 5). As shown in Figure 5, *SiROS1d* was highly expressed in all tissues, while *SiDML3b* and *SiDML4* were almost undetected in all tissues. *SiROS1b*, *SiDML3a*, *SiDML5* and *SiROS1a* were highly expressed in most tissues. *SiDML3a*, *SiDML5* and *SiROS1a* were all highly expressed in leaf 2–5, whereas the expression of the other genes was low, suggesting that these three genes may have similar functions in leaf development. *SiDML3a* was highly expressed in total aerial, indicating that probably *SiDML3a* has an important function in plant growth and development. Consistent with these results, the promoters of these genes have light-responsive regulatory elements such as AE-box, GT1-motif and TCT-motif. The expression of *SiROS1d*, *SiROS1b*, *SiDML3a*, *SiDML5* and *SiROS1a* was higher in roots during ammonia, drought, nitrate, and urea treatments, suggesting that these genes may be involved in plant adversity stress. The analysis of cis-acting elements also showed that the promoters of these genes have multiple stress responses regulatory elements, such as MBS involved in drought induction and TC-rich repeats involved in defense and stress responses. These results suggest that the DNA-deMTase genes play regulatory roles at different stages of plant growth and development and in different tissues. 

### 2.7. The Transcript Abundance of DNA-deMTase Genes in S. italica under Abiotic Stress

To clarify the potential roles of DNA-deMTase genes involved in abiotic stress, we used qRT-PCR to determine the abundance of DNA-deMTase genes in the transcript under cold, drought, NaCl, ABA, and MeJA stresses. 

**Figure 6:**
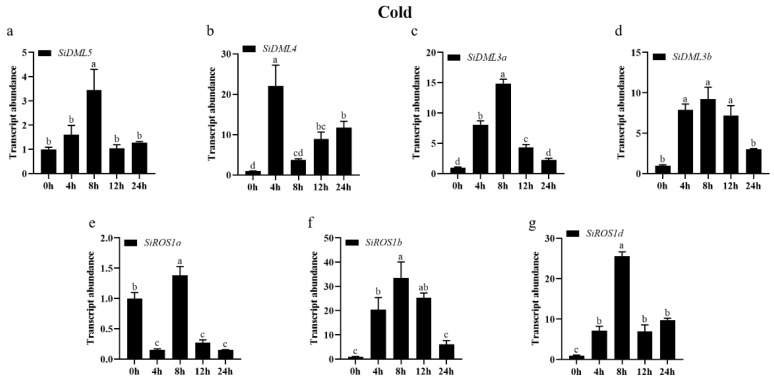
Transcript abundance of *SiDML5* (**a**), *SiDML4* (**b**), *SiDML3a* (**c**), *SiDML3b* (**d**), *SiROS1a* (**e**), *SiROS1b* (**f**), *SiROS1d* (**g**) under cold stress. Data are the mean ± SE (*n* = 3). Different letters above bars indicate statistically significant differences between treatments (Tukey’s multiple range test, *p* ≤ 0.05).

The transcript abundance of all DNA-deMTase genes changed significantly under cold treatment conditions (Figure 6). The expression patterns of *SiDML5*, *SiDML4*, *SiDML3a*, *SiDML3b*, *SiROS1b*, and *SiROS1d* were consistent with a tendency to increase and then decrease during the 24 h of cold stress. Among them, the expression of *SiDML5* was significantly increased at 8 h, the expression of other genes was significantly increased at 4 h. The expression of *SiROS1a* was first decreased and then increased after the cold stress treatment, and then decreased again. The expression of *SiROS1a* was significantly increased at 8 h, and was significantly lower than 0 h at all the other times. 

**Figure 7:**
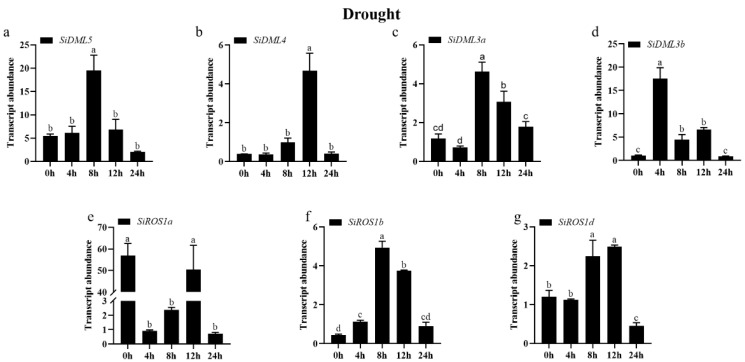
Transcript abundance of *SiDML5* (**a**), *SiDML4* (**b**), *SiDML3a* (**c**), *SiDML3b* (**d**), *SiROS1a* (**e**), *SiROS1b* (**f**), *SiROS1d* (**g**) under drought stress. Data are the mean ± SE (*n* = 3). Different letters above bars indicate statistically significant differences between treatments (Tukey’s multiple range test, *p* ≤ 0.05).

The transcript abundance of all DNA-deMTase genes changed significantly under drought treatment conditions (Figure 7). The expression patterns of *SiDML5*, *SiDML4*, *SiDML3a*, *SiDML3b*, *SiROS1b*, and *SiROS1d* were consistent with a tendency to increase and then decrease during the 24 h of drought stress. Among them, the expression of *SiDML4* was significantly increased at 12 h, the expression of *SiDML3b* was significantly increased at 4 h, and the expression of other genes was significantly increased at 8 h. However, not all DNA-deMTase genes responded to drought stress, and the expression of *SiROS1a* was first decreased and then increased after the cold stress treatment, and then decreased again., but was always lower than 0 h. 

**Figure 8:**
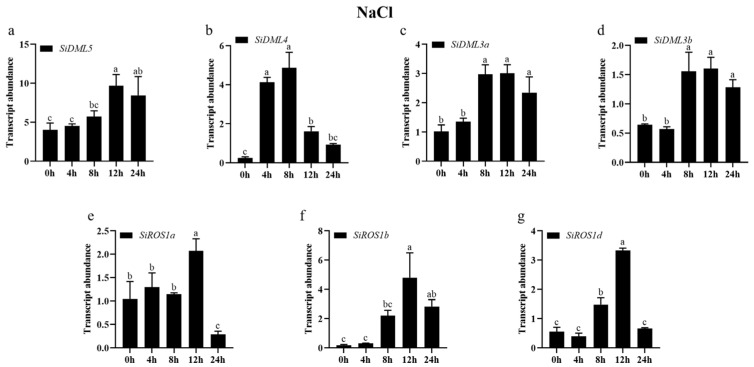
Transcript abundance of *SiDML5* (**a**), *SiDML4* (**b**), *SiDML3a* (**c**), *SiDML3b* (**d**), *SiROS1a* (**e**), *SiROS1b* (**f**), *SiROS1d* (**g**) under NaCl stress. Data are the mean ± SE (*n* = 3). Different letters above bars indicate statistically significant differences between treatments (Tukey’s multiple range test, *p* ≤ 0.05).

The transcript abundance of all DNA-deMTase genes changed significantly under NaCl treatment conditions (Figure 8). The expression patterns of *SiDML5*, *SiDML3a*, and *SiDML3b* were consistent with a tendency to increase during the 24 h of NaCl stress. Among them, the expression of *SiDML5* was significantly increased at 12 h, and the expression of other genes was significantly increased at 8 h. The expression patterns of *SiDML4*, *SiROS1a*, *SiROS1b*, and *SiROS1d* were consistent with a tendency to increase and then decrease during the 24 h of NaCl stress. The expression of *SiDML4* was significantly increased at 4 h, and the expression of other genes was significantly increased at 12 h. The expression of *SiDML4* was significantly increased at 4 h, the expression of *SiROS1d* was significantly increased at 8 h, and the expression of other genes was significantly increased at 12 h. 

**Figure 9:**
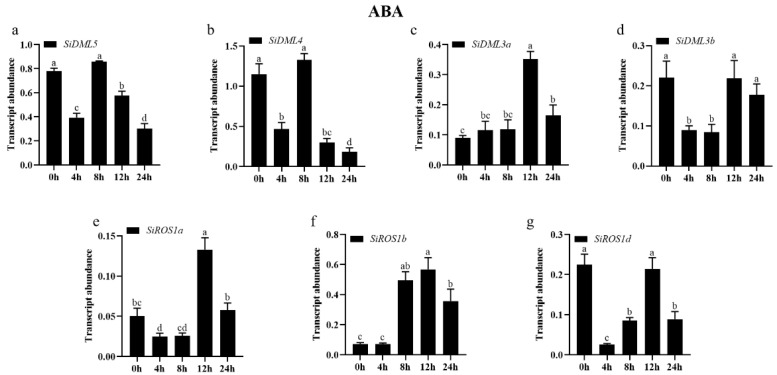
Transcript abundance of *SiDML5* (**a**), *SiDML4* (**b**), *SiDML3a* (**c**), *SiDML3b* (**d**), *SiROS1a* (**e**), *SiROS1b* (**f**), *SiROS1d* (**g**) under ABA stress. Data are the mean ± SE (*n* = 3). Different letters above bars indicate statistically significant differences between treatments (Tukey’s multiple range test, *p* ≤ 0.05).

The transcript abundance of all DNA-deMTase genes changed significantly under ABA treatment conditions (Figure 9). The expression patterns of *SiDML3a* and *SiROS1b* were consistent with a tendency to increase and then decrease during the 24 h of ABA stress. Furthermore, the expression of *SiDML3a* and *SiROS1b* was significantly increased at 12 h; the expression of *SiROS1a* was first decreased and then increased after the ABA stress treatment, and then decreased again. However, not all DNA-deMTase genes responded to ABA stress. The expression of *SiDML5*, *SiDML4*, and *SiROS1d* was first decreased and then increased after the ABA stress treatment, and then decreased again, but was always lower than 0 h; the expression of *SiDML3b* was first decreased and then increased after the ABA stress treatment but was always lower than 0 h. 

**Figure 10:**
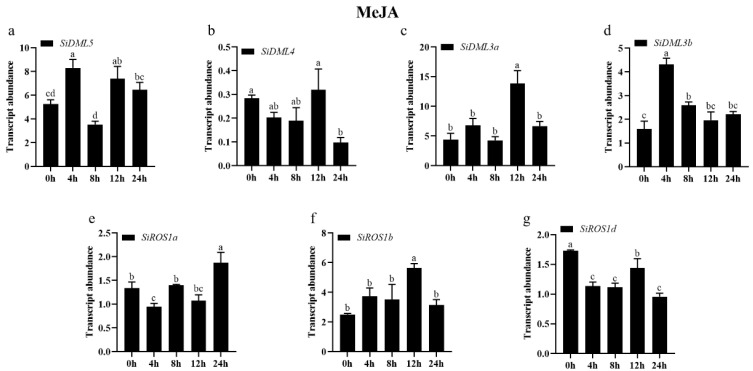
Transcript abundance of *SiDML5* (**a**), *SiDML4* (**b**), *SiDML3a* (**c**), *SiDML3b* (**d**), *SiROS1a* (**e**), *SiROS1b* (**f**), *SiROS1d* (**g**) under MeJA stress. Data are the mean ± SE (*n* = 3). Different letters above bars indicate statistically significant differences between treatments (Tukey’s multiple range test, *p* ≤ 0.05).

The transcript abundance of all DNA-deMTase genes changed significantly under MeJA treatment conditions (Figure 10). The expression patterns of *SiDML3a*, *SiDML3b* and *SiROS1b* were consistent with a tendency to increase and then decrease during the 24 h of MeJA stress. Furthermore, the expression of *SiDML3a* and *SiROS1b* was significantly increased at 12 h, the expression of *SiDML3b* was significantly increased at 4 h; the expression of *SiDML5* was first increased and then decreased after the MeJA stress treatment, and then increased again; the expression of *SiROS1a* were consistent with a tendency to decrease and then increase during the 24 h of MeJA stress. However, not all DNA-deMTase genes responded to MeJA stress, and the expression of *SiROS1d* was first decreased and then increased after the MeJA stress treatment, and then decreased again, but was always lower than 0 h; the expression of *SiDML4* was decreased after the MeJA stress treatment. 

Temperature, drought, and salt stress are the main abiotic stresses in agriculture worldwide that threaten the growth and development of *S. italica*. ABA and MeJA as stress signaling factors for adversity can also improve plant stress tolerance. The above results suggest that DNA-deMTase genes from *S. italica* play important roles in abiotic stresses.

## 3. Discussion

### 3.1. Structural Features of DNA-deMTase in S. italica

With the release of the *S. italica* reference genome, the identification, classification, and prediction of gene family functions have gradually become new research hotspots. To elucidate the regulatory role of DNA demethylation in *S. italica*, we identified the DNA-deMTase genes at the whole genome level. The cis-acting elements, conserved motifs, phylogenetic relationships, and sequence features of these genes were also integratively analyzed. Currently, DNA-deMTase genes have been characterized in a variety of plants, such as *A. thaliana*, *Solanum lycopersicum*, *Ricinus communis*, *Fragaria vesca*, peanut [50,51,52,53,54], cotton and other plants. In this study, seven DNA-deMTases from *S. italica* were identified.

To identify factors in *S. italica* where specific epigenetic genes can regulate transcription, the promoter sequences of their putative cis-acting elements within 2000 bp upstream of the gene were analyzed. The results showed that the promoters of DNA-deMTases have four types of cis-actors: light response, phytohormone response, stress response, and growth and development. Among them, the stress-responsive elements are mainly involved in low-temperature stress, drought-induced, and stress responses. Similar CREs have been reported in the promoter regions of the DNA-deMTase gene of other plants such as Dendrobium officinale, tea plant, and kiwifruit [55,56,57].

### 3.2. Expression of the DNA-deMTase Gene in Abiotic Stress

Increasing evidence from recent studies suggests that DNA methylation plays an important role in regulating the stress response in plants [58,59], and change under different environmental stresses [60,61]. It was found that salt stress inhibited germination and seedling growth of *S. italica* seeds, and the restrain on radicel was bigger than that of on plantule, the relative radicel length was longer than relative plantule length [62]. Drought stress inhibited the rate of superoxide generation and hydrogen peroxide accumulation in *S. italica* seedlings, while decreasing MDA content [63]. *SiARDP* is a DREB-type transcription factor from *S. italica*. The transcript levels of *SiARDP* increased not only after drought, high salt, and low temperature stresses, but also after an ABA treatment of *S. italica* seedlings, indicating a key role in plant abiotic stress response [64]. Therefore, uncovering the mechanisms of tolerance to abiotic stress in plants and searching for related functional genes are essential tasks for successful molecular breeding. To adapt to environmental challenges, plants can make adjustments in response to various abiotic and biotic stresses [65,66]. Research has found that drought stress is associated with changes in genomic methylation of many genes, including those encoding transcription factors. In poplar, transcription factors that affect gene expression after drought treatment are influenced by methylated transposons [67]. Under salt stress, DNA methylation can control stress signals and induce plant stress responses by regulating membrane transporter genes, heavy metal transporter genes, and organic acid secretion genes. Under cold stress, genes involved in cellular metabolism, the stress response, the antioxidant system, the lysine metabolism pathway, and transcriptional regulation exhibit a correlation between methylation and their expression [68,69]. In addition, when plants are exposed to abiotic stress, the level of DNA methylation changes, and this effect can be repeatedly established [70].

In this study, we investigated the abundance of DNA-deMTase gene transcripts under cold, drought, salt, ABA, and MeJA stress. The results showed that the abundance of transcripts from all DNA-deMTase genes in *S. italica* under abiotic stress showed a dynamic trend at specific times from 0 to 48 h. These results suggest that gene families play different roles in abiotic stress, suggesting that demethylation of some key genes may be crucial for abiotic responses in plants.

## 4. Materials and Methods

### 4.1. Identification of DNA Demethylase Proteins in S. italica

It was shown that there are four DNA demethylase genes in the *Arabidopsis thaliana* dicotyledons plant: *ROSl*, *DME*, *DML2* and *DML3* [71], and eight DNA demethylase genes in the monocotyledon plant *Oryza sativa*: *ROSla*, *ROSlb*, *ROSlc*, *ROS1d*, *DML3a*, *DML3b*, *DML4* and *DML5* [72]. The amino acid sequences of DNA demethylases of *A. thaliana*, *O. sativa*, and *Triticum aestivum* [71,72,73] were used as queries in BLASTp (E value > 10^−5^) searches against the *S. italica* annotation database. To confirm the precision of these predicted genes, amino acid sequences were analyzed using CDD and Smart (http://smart.embl-heidelberg.de/, accessed on 20 August 2023), and sequences without conserved domains were excluded. *S. italica* genome information was downloaded from Phytozome v13 (https://phytozome-next.jgi.doe.gov/, accessed on 17 August 2023) [74]. The homologous DNA demethylase proteins in *A. thaliana* were obtained from TAIR (https://www.arabidopsis.org/, accessed on 20 August 2023); the homologous DNA demethylase proteins in *O. sativa* v7.0 were obtained from Phytozome v13 (https://phytozome-next.jgi.doe.gov/, accessed on 20 August 2023); the homologous DNA demethylase proteins in *Triticum aestivum* (IWGSC) were obtained from Ensembl Plants (https://plants.ensembl.org/index.html, accessed on 20 August 2023). The Plant-mPLoc 2.0 (http://www.csbio.sjtu.edu.cn/bioinf/plant-multi/, accessed on 25 September 2023) was used to predict the subcellular localization of these genes, and the ProtParam program (http://web.expasy.org/protparam/, accessed on 30 September 2023) in ExPASy was used to obtain the theoretical molecular weight (MW) and isoelectric point (pI), etc. of these proteins.

### 4.2. Phylogenetic Tree and Evolutionarily Conserved Protein Domain Analysis 

The phylogenetic tree was established by 1000 bootstrap replications using Molecular Evolutionary Genetics Analysis (MEGA 7.0.26) software, based on the neighbor-joining (NJ) method [75,76]. DNA-deMTase were classified based on their phylogenetic relationship with the corresponding DNA-deMTase proteins from *A. thaliana*, *O. sativa*, and *T. aestivum*, and genes were named according to the chromosomal location of *S. italica* DNA-deMTase. The evolutionary tree was beautified by ITOL V6 (https://itol.embl.de, accessed on 20 August 2023) online software. The amino acid sequences and conserved domains of these proteins were analyzed using a conserved domain database (https://www.ncbi.nlm.nih.gov/Structure/cdd/wrpsb.cgi, accessed on 20 August 2023) [77].

### 4.3. Conserved Motifs and Gene Structure

The conserved motifs of all DNA-deMTase proteins were analyzed using Multiple Expectation Maximization for Motif Elicitation (MEME Suite 5.5.5, http://meme-suite.org/, accessed on 23 August 2023) by uploading the amino acid sequences following the MEME instructions. The conserved motif sites were visualized using TBtools v2.042 [78]. Gene structure Display Server 2.0 (https://gsds.gao-lab.org/, accessed on 23 August 2023) [79] was used to show the exon/intron structure of each DNA demethylase gene.

### 4.4. Analysis of Cis-Regulatory Elements and Chromosome Localization

The upstream sequences (2000 bp) of the start codon were retrieved from the *S. italica* genome database. Then the types, numbers, and functions of promoter cis-acting elements were analyzed by PlantCARE (http://bioinformatics.psb.ugent.be/webtools/plantcare/html/, accessed on 28 August 2023) and visualized by TBtools v2.042 [80]. The locations of the genes identified on the chromosomes of *S. italica* were taken from the GFF (Graphics File Formats) file, which was downloaded from the genome database. The physical map of the chromosome was visualized by TBtools v2.042.

### 4.5. Expression of DNA Demethylases

FPKM (Fragments Per Kilobase of exon model per Million fragments mapped) expression value data (RNA-seq) of DNA demethylase genes of 7 tissues at different developmental stages of cereal were obtained from phytozome, and the FPKM values can reflect the gene expression level. The heatmap of gene expression was obtained using the TBtools v2.042.

### 4.6. Plant Material, Growth Condition and Abiotic Stress Treatment

The seeds of *Setaria italica* L. (*Xiaomi*) were obtained from Prof. Xingchun Wang, The Shanxi Agricultural University, China. Seeds were incubated in water for one night in the dark, evenly spread on beakers covered with gauze filled with water, and incubated in the dark for three days at 28 °C, replenishing water every six hours. Then, the uniformly grown cereal seedlings were selected and evenly placed in 96-well hydroponic boxes and cultured in Hoagland nutrient solution, which was changed every three days. The plants were placed in a plant growth chamber at 23 °C, bright fluorescent light of 150 µmol photons m^−2^ s^−1^, relative humidity of 65% and 16 h light: 8 h dark cycle.

For stress treatment, 30-day-old seedlings were subjected to six abiotic stresses, namely, cold (4 °C) [81], drought (20% PEG6000) [82], salt (150 Mm NaCl), ABA (100 µmol) [83], and MeJA (100 µmol) for a duration of 0, 4, 8, 12, and 24 h in triplicates. After 8 h of salt stress, the leaves began to wilt; at 12 h, more than half of the leaves had wilted; at 24 h, the plant wilted. No phenotypes were observed during the sampling of other treatments.

### 4.7. Total RNA Extraction and Transcript Abundance Analyzes of DNA-deMTase Genes

Total RNA from *S. italica* leaves was extracted using TransZol Up reagent (TransGen Biotech, Beijing, China). Total RNA was then reverse transcribed into the first strand cDNA using an *Easyscript* RT/RI Enzyme Mix (TransGen Biotech, Beijing, China). Quantitative real-time polymerase chain reaction (qRT-PCR) was performed with 2×PerfectStart Green qPCR SuperMix (TransGen Biotech, Beijing, China). The abundance of transcripts was calculated using the 2^−ΔΔCt^ method [84], and all primers used for qRT-PCR were designed using NCBI, and both the qRT-PCR procedure and the reaction system were used in Table S1. All qRT-PCR analyzes were performed in three biological replicates. Statistical analyses were performed using IBM SPSS Statistics 21 software.

## 5. Conclusions

According to the reference genome of *S. italica*, seven DNA-deMTase genes were discovered and identified. Phylogenetic analysis demonstrated that the seven DNA-deMTase genes were divided into four categories, DML5, DML4, DML3, and ROS1. The DNA-deMTase promoter has a variety of stress-responsive, hormone signaling, and plant development-related regulatory elements, and DNA-deMTase genes play a regulatory role in different stages of plant growth and development and in different tissues. This indicates that the demethylase family plays an important role in the growth and development of *S. italica*. To gain further insight into the potential roles of DNA-deMTase involved in stress response, we analyzed seven DNA-deMTase genes from *S. italica.* Under cold and salt stress, all DNA-deMTase genes were induced with noticeably higher expression levels compared with the control (0 h). Under drought stress, *SiDML5*, *SiDML4*, *SiDML3a*, *SiDML3b*, *SiROS1b*, and *SiROS1d* were highly expressed compared with the control at 8 h and 12 h, while *SiROS1a* was lowly expressed. Under ABA treatments, the majority of *SiDML3a*, *SiROS1a*, and *SiROS1b* were upregulated. Among these genes, *SiROS1b* was the most preferentially expressed in response to ABA treatment. Additionally, *SiDML5*, *SiDML3a*, *SiDML3b*, *SiROS1a*, and *SiROS1b* were induced with higher expression levels under MeJA stress treatments. The results of this study lay the foundation for the discovery of functional genes and the further study of the stress resistance mechanisms of *S. italica*.

## Figures and Tables

**Table 1:** Characteristic features of DNA demethylase (DNA-deMTase) gene family in Foxtail millet (*Setaria italica* L.).

Gene Name	Gene ID	No. of Introns	Length (Aa)	MW (kDa)	pI	Instability Index	GRAVY Value	Subcellular Localization
*SiDML5*	*Seita.8G104300*	10	358	39.74	9.67	41.68	−0.455	Chloroplas. Nucleus.
*SiDML3a*	*Seita.1G164500*	16	1060	118.04	7.84	49.08	−0.549	Nucleus.
*SiROS1b*	*Seita.3G223900*	19	1663	185.59	6.20	56.29	−0.640	Nucleus.
*SiROS1a*	*Seita.2G152900*	17	2082	230.61	6.79	46.03	−0.605	Nucleus.
*SiROS1d*	*Seita.1G164200*	16	1954	216.94	6.45	47.77	−0.672	Nucleus.
*SiDML3b*	*Seita.9G185500*	11	578	65.77	5.97	42.44	−0.665	Nucleus.
*SiDML4*	*Seita.4G105300*	4	286	31.52	9.10	56.21	−0.300	Nucleus.

Aa: amino acid; MW: Molecular weight; pI: theoretical isoelectric point; GRAVY: Grand average of hydropathy.

## Data Availability

The materials and datasets generated during and/or analyzed during the current study are available from the corresponding author on reasonable request.

## Supplementary Materials

The following supporting information can be downloaded at: https://www.mdpi.com/article/10.3390/ijms25084464/s1.
