# Supplementary material for: Systematic Analysis of DNA Demethylase Gene Families in Foxtail Millet (Setaria italica L.) and Their Expression Variations after Abiotic Stresses
Source: Int J Mol Sci. 2024 Apr 18;25(8):4464. doi: 10.3390/ijms25084464 (PMC11050331; doi:10.3390/ijms25084464)
Supplement: Supplementary file 1 [file ijms-25-04464-s001.zip › ijms-2936320-supplementary.pdf]

Supplementary Materials

Table. S1 Primers used for RT-qPCR analysis in this study

Gene	Specific Primer
SiDML5	F:5'- GGATGAGTGGGAACCCATAAA - 3'<br>R:5'- GCTGAAGGGCAAAGGTTATTG -3'
SiDML4	F:5'- AGGCTATGGGTTGGGTTCCT -3'<br>R:5'- CCTTCTCCCTGGTACATGTTG -3'
SiDML3a	F:5'- AGCCATTAATGATGCCTCCCA -3'<br>R:5'- GCAATCGGTGCGTGTAGTA -3'
SiDML3b	F:5'- CAGACCGGGTACTAAACAGATG -3'<br>R:5'- GGAGTTTCTGGTTCCTGGATAA -3'
SiROS1a	F:5'- ATCTTTCCAGCTCTGCCTTTAT -3'<br>R:5'- CTCGAGCAGCAACCATTATCT -3'
SiROS1b	F:5'- TTGTCTGTGTGCGAGGATTT -3'<br>R:5'- TGCTGTCTTGCGGGATTT -3'
SiROS1d	F:5'- CATCGATCCAGTCACCACTATG -3'<br>R:5'- CTTCGTCAAGCCATTTCTCTTTATC -3'
Actin	F:5'- CGCATATGTGGCTCTTGACT-3'<br>R:5'- GGGCACCTAAATCTCTCTGC-3'

Gene-specific primers for real-time reverse transcription quantitative PCR (RT-qPCR) were designed by the Primer Quest tool (http://www.idtdna.com/Primerquest/Home/Index). F, forward; R, reverse.

Table. S2 Information of DNA demethylase genes in the 3 tested species

Latin name of specie	Gene name	Gene ID
Arabidopsis thaliana	*AtDML5a*	*AT2G31450.1*
	AtDML5b	*AT1G05900.1*
	AtDML4	*AT3G47830.1*
	AtDML3	*AT4G34060.3*
	AtDML2	*AT3G10010.1*
	AtDME	*AT5G04560.1*
	AtROS1	*AT2G36490.1*
Oryza sativa	*OsDML5*	*LOC_Os11g16580.1*
	OsDML4	*LOC_Os06g13070.1*

	OsDML3a	*LOC_Os02g29380.1*
	OsDML3b	*LOC_Os04g28860.1*
	OsROS1a	*LOC_Os01g11900.1*
	OsROS1b	*LOC_Os05g37350.1*
	OsROS1c	*LOC_Os05g37410.1*
	OsROS1d	*LOC_Os02g29230.1*
Triticum aestivum	*TaROS1b-1A.1*	*TraesCS1A02G278000.1*
	TaROS1b-1B.1	*TraesCS1B02G286900.2*
	TaROS1b-1D.1	*TraesCS1D02G277100.1*
	TaROS1b-1A.2	*TraesCS1A02G349600.1*
	TaROS1b-1B.2	*TraesCS1B02G364100.2*
	TaROS1b-1D.2	*TraesCS1D02G352500.2*
	TaDML3a-3A	*TraesCS3A02G022500.1*
	TaDML3a-3B	*TraesCS3B02G023200.1*
	TaDML3a-3D	*TraesCS3D02G024100.1*
	TaDML5-4A	*TraesCS4A02G232700.2*
	TaDML5-4B	*TraesCS4B02G083100.1*
	TaDML5-4D	*TraesCS4D02G081200.1*
	TaROS1a-5A	*TraesCS5A02G169000.1*
	TaROS1a-5B	*TraesCS5B02G165800.2*
	TaROS1a-5D	*TraesCS5D02G173300.2*
	TaDML4-7A	*TraesCS7A02G194800.1*
	TaDML4-7B	*TraesCS7B02G100500.1*
	TaDML4-7D	*TraesCS7D02G196400.1*
